# Maturation, Behavioral Activation, and Connectivity of Adult-Born Medium Spiny Neurons in a Striatal Song Nucleus

**DOI:** 10.3389/fnins.2017.00323

**Published:** 2017-06-07

**Authors:** Jennifer Kosubek-Langer, Lydia Schulze, Constance Scharff

**Affiliations:** Animal Behavior, Freie Universität BerlinBerlin, Germany

**Keywords:** adult neurogenesis, songbird, basal ganglia, Area X, EGR-1, DARPP-32, dopamine

## Abstract

Neurogenesis continues in the adult songbird brain. Many telencephalic song control regions incorporate new neurons into their existing circuits in adulthood. One song nucleus that receives many new neurons is Area X. Because this striatal region is crucial for song learning and song maintenance the recruitment of new neurons into Area X could influence these processes. As an entry point into addressing this possibility, we investigated the maturation and connectivity within the song circuit and behavioral activation of newly generated Area X neurons. Using BrdU birth dating and virally mediated GFP expression we followed adult-generated neurons from their place of birth in the ventricle to their place of incorporation into Area X. We show that newborn neurons receive glutamatergic input from pallial/cortical song nuclei. Additionally, backfills revealed that the new neurons connect to pallidal-like projection neurons that innervate the thalamus. Using *in situ* hybridization, we found that new neurons express the mRNA for D1- and D2-type dopamine receptors. Employing DARPP-32 (dopamine and cAMP-regulated phosphoprotein of 32 kDa) and EGR-1 (early growth response protein 1) as markers for neural maturation and activation, we established that at 42 days after labeling approximately 80% of new neurons were mature medium spiny neurons (MSNs) and could be activated by singing behavior. Finally, we compared the MSN density in Area X of birds up to seven years of age and found a significant increase with age, indicating that new neurons are constantly added to the nucleus. In summary, we provide evidence that newborn MSNs in Area X constantly functionally integrate into the circuit and are thus likely to play a role in the maintenance and regulation of adult song.

## Introduction

Adult neurogenesis is an enigmatic trait. Only some neurons continue to be generated in adulthood whereas the majority are born during development and persist throughout the animal's life. Why these differences exist is still not known but much progress has been made elucidating the mechanism and function of adult neurogenesis during the past decades (Song et al., 2016). Neurons born in adulthood originate in regions adjacent to the ventricles that also give rise to neurons during development. From these neurogenic niches, neural precursors delaminate and then migrate through the dense parenchyma, incorporate into functional circuits and influence behavior (Paredes et al., 2016).

Considerable differences exist with respect to the extent of adult neurogenesis in different species. As a rule of thumb, adult-born new neurons are recruited to many brain regions in vertebrates like teleost fish, amphibians, and reptiles, whereas in birds the extent is still widespread but more restricted to the forebrain (Kaslin et al., 2008). In mammals, there are even fewer regions that continue to recruit new neurons in adulthood, principally the dentate gyrus (DG) of the hippocampal formation (Kempermann et al., 2015) and the olfactory bulb (Lim and Alvarez-Buylla, 2016). Interestingly, in rats, rabbits, monkeys and humans but not in mice, adult-generated neurons have also been observed in the striatum (Bedard et al., 2002; Dayer et al., 2005; Tonchev et al., 2005; Luzzati et al., 2006; Ernst et al., 2014). In these cases, the newly generated neurons belong primarily to the class of GABAergic interneurons, which constitute less than 5% of the striatal neurons (Tepper et al., 2010). The most abundant striatal cell type are medium spiny projection neurons (MSNs) (Gerfen and Wilson, 1996). In adult rodents, generation of MSNs has only been reported in response to experimentally induced stroke, ischemia, or lesions (Arvidsson et al., 2002; Tattersfield et al., 2004; Hou et al., 2008). In contrast, in songbirds adult MSNs keep immigrating in substantial numbers into the striatum under natural conditions (Alvarez-Buylla et al., 1990). Striatal newborn neurons originate from the progenitor containing subpallial region in the lateral ventricle that expresses the transcription factors ISL-1/2, NKX2.1, and DLX but not TBR1 (Scott and Lois, 2007). Of particular interest is the recruitment of MSNs into Area X (Nordeen and Nordeen, 1988; Rochefort et al., 2007; Scott and Lois, 2007) a region unique to songbirds relevant for song plasticity in juveniles and adults (Sohrabji et al., 1990; Scharff and Nottebohm, 1991; Jarvis et al., 1998; Hessler and Doupe, 1999; Woolley et al., 2014). In songbirds, new neurons destined for Area X migrate between 1,000 and 2,000 μm to their final destination.

**Figure 1:**
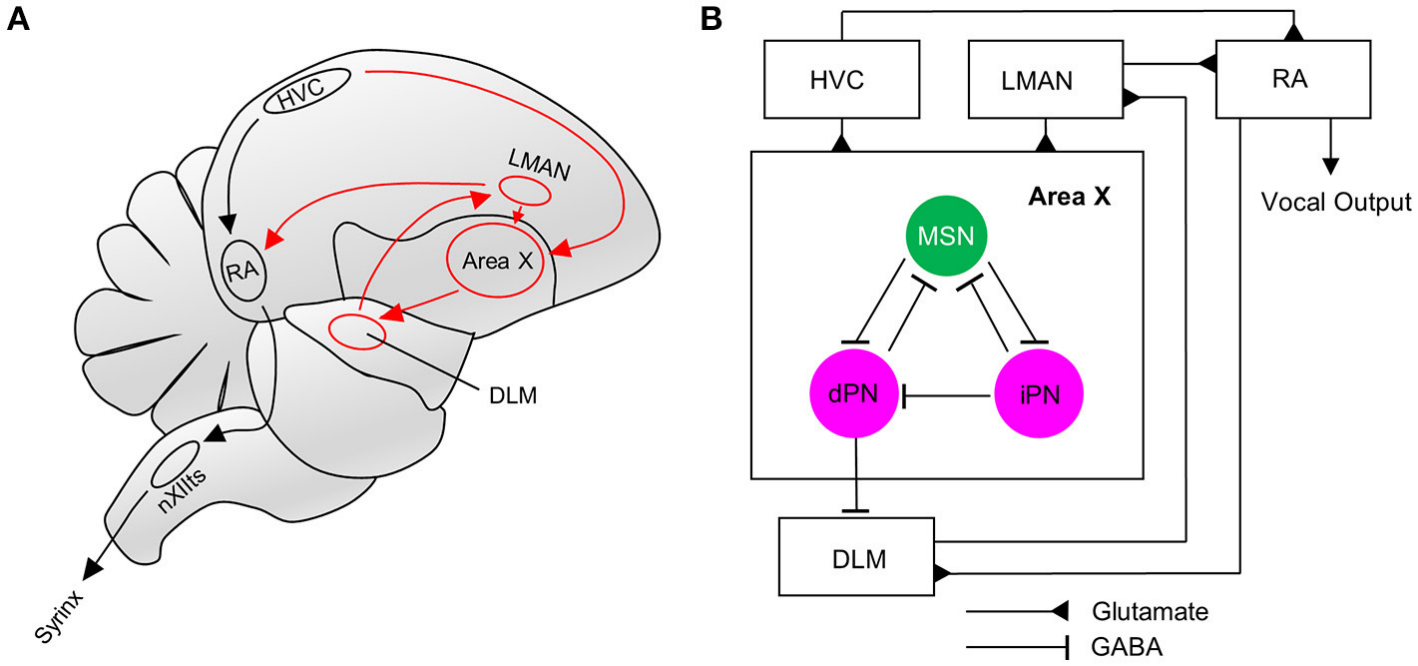
The song system and connectivity within Area X. **(A)** The song motor pathway (shown in black) controls the vocal organ (syrinx) via HVC->RA->nXIIts. The anterior forebrain pathway (AFP, shown in red) forms a cortico-basal ganglia-thalamic-cortical loop, connecting HVC and RA via Area X->DLM->LMAN. **(B)** Neurons in Area X receive glutamatergic innervations from HVC and LMAN. MSNs in Area X inhibit direct and indirect pallidal-like neurons (dPN, iPN). Both types can project to MSNs, but only dPNs project to the thalamic nucleus DLM that connects to RA via LMAN (Farries et al., 2005; Goldberg et al., 2010). RA directly innervates the AFP via DLM. RA, Robust nucleus of the arcopallium; LMAN, Lateral magnocellular nucleus of the anterior nidopallium; XII, Nucleus; NXIIts, tracheosyringeal part; DLM, Dorsal lateral nucleus of the medial thalamus.

The dynamics of neural recruitment are best understood in the DG and the olfactory bulb. In the former, new neurons are added, whereas in the latter, they replace older neurons that undergo apoptosis (Crespo et al., 1986; Imayoshi et al., 2008). In both cases, the time it takes for new neurons to incorporate into preexisting circuits is similar (Deshpande et al., 2013). In songbirds, the dynamics of neural recruitment have only been studied in the pallial/cortical song control region HVC (proper name, Figure 1A), where glutamatergic projection neurons undergo neurogenesis (Kirn et al., 1999; Scott and Lois, 2007; Tokarev et al., 2016).

To gain insight into the integration of GABAergic MSNs into existing circuits, we studied their differentiation, connectivity and activation by singing in Area X. To do so we traced new neurons by injections of green fluorescent protein (GFP)-expressing lentivirus into the lateral wall of the lateral ventricle and with systemic injections of the cell birth marker 5-bromo-2′-deoxyuridine (BrdU). We also injected retrograde tracer into one of the target regions of Area X, and used immuno- and *in situ*- histochemistry to characterize the new neurons. We report that adult born MSNs receive glutamatergic and dopaminergic input, connect to pallidal-like projection neurons and are activated during singing like older, resident MSNs.

Because new HVC neurons seem to replace older ones in canaries (Kirn and Nottebohm, 1993), whereas in zebra finches constant neuronal addition was observed (Walton et al., 2012) we also addressed the issue of replacement vs. addition. We quantified neuron numbers in adult zebra finches of varying age and found that the density of MSNs in Area X increased with age, supporting the idea of neuron addition rather than replacement. Overall, our results suggest that Area X receives a constant addition of functional new GABAergic MSNs.

## Materials and methods

### Animals

Adult male zebra finches (*Taeniopygia guttata*) were bred and housed at the Department of Animal Behavior at Freie Universität Berlin. The colony was kept under a 12:12 h light:dark-cycle and food and water were available *ad libitum*. All procedures were reviewed and approved by the veterinary department of the Freie Universität Berlin and by the ethics committee of the Regional Office for Health and Social Affairs Berlin (LAGeSo). The permit numbers are G0116/13 and G0296/15. In total, we used 53 adult male zebra finches. For the expression analysis of the early growth response protein 1 (EGR-1) and the dopamine- and cAMP-regulated neuronal phosphoprotein (DARPP-32) in newborn cells we used 29 birds (age 462 ± 158 days, mean ± standard deviation, SD). Dopamine (DA) receptor expression was studied in 5 birds (age 172 days ± 13 days, mean ± SD). Five birds received lentiviral injections (age 367 days ± 109 days, mean ± SD). Density measures in Area X were performed in 14 birds (age ranging from 372 to 2,526 days).

### BrdU injections

Birds for EGR-1 and DA receptor analysis received BrdU (50 μg/g) via intramuscular injections in the mornings for 5 consecutive days. Birds were assigned to three groups with different survival times after BrdU injection (21, 31, and 42 days). We choose the first survival time to be 21 days, because BrdU+ neurons in Area X were previously shown to express immediate early genes after singing at that time (Tokarev et al., 2016).

### Song monitoring

For subsequent EGR-1 analysis, birds were kept in sound attenuated chambers for three nights and were perfused in the morning of the 4th day 1.5 h after the lights went on. Vocalizations were continuously monitored via Sound Analysis Pro (Tchernichovski et al., 2000). During those 1.5 h birds had to sing at least 150 motifs to be included in the subsequent analysis of EGR-1 expression.

Birds that received lentiviral injections and retrograde tracer were isolated in sound attenuated chambers for one night before sacrifice. Birds were kept from singing by the experimenter sitting nearby for 1.5 h after lights went on in the morning and then killed. This was necessary because we used some of the brain sections in another experiment to be reported elsewhere.

Birds used for DA receptor analysis were decapitated without previous song monitoring and their brains were quickly dissected 1.5 h after the lights went on. All birds were killed by isoflurane overdose.

### Lentiviral vector injection and backfill

To label progenitors in the lateral wall of the ventricle, the lentiviral expression vector pFUGW (Lois et al., 2002) containing a GFP reporter gene was stereotactically injected into the ventricular zone under isofluorane anesthesia. Birds were fixed in a stereotaxic head holder, with the beak in a 45° angle from the vertical axis. In each hemisphere, we injected four sites with approximately 200 μl of viral construct using the following coordinates relative to the bifurcation of the midsagittal sinus: anterior-posterior 3.8–4.1, medial-lateral −1.3/+1.3, dorsal-ventral −5.0, injection angle AP 10°. To label pallidal-like projection neurons, we injected approximately 600 μl tetramethylrhodamine coupled with biotin (BDA, 3,000 MW, Molecular Probes) into DLM 4–5 days before sacrifice at day 42. We used the following coordinates: anterior-posterior 1.2, medial-lateral −1.3/+1.3, dorsal-ventral −4.5. After surgeries birds were transferred to their home cages. To confirm that the virus infected proliferating cells, some birds were injected with BrdU (50 μg/g) on the day of surgery.

### Immunohistochemistry and image analysis

For immunohistochemistry birds were overdosed with isoflurane and then perfused transcardially with phosphate-buffered saline (PBS) followed by 4% paraformaldehyde (PFA) in PBS. After dissection, brains were post-fixed for one night, washed for another night in PBS and cut sagitally or coronally into 50 μm sections using a vibrating microtome (VT1000S, Leica). For BrdU antigen retrieval, sections were incubated in 2 N HCl for 30 min at 37°C and neutralized with borate buffer. All other immunostainings were performed according to standard protocols. The following antibodies were used; primary: anti EGR-1 (rabbit, Santa Cruz sc-189), anti DARPP-32 (mouse, kindly provided by H.C. Hemmings, Jr., Weill Cornell Medical College, New York), anti DARPP-32 (rabbit, abcam ab40801), anti BrdU (rat, Bio-Rad MCA2060), anti VGLUT2 (mouse, abcam ab79157), anti GFP (rabbit, abcam ab290). Fluorescent Secondary: anti-rabbit-Alexa-Fluor-568 (life technologies, A10042), anti-mouse-Alexa-Flour-568 (Life technologies, A10037), anti-rat-Alexa-Fluor-488 (Life Technologies, A21208), anti-rabbit-Alexa-Fluor-488 (Life Technologies, A21206). Biotinylated dextran signal was amplified using Streptavidin-Alexa-Fluor-568 (Life Technologies, S11226). Sections were counterstained with 4′,6-Diamidin-2-phenylindol (DAPI, Serva). Z-Stacks were obtained with a SP8 confocal microscope (Leica) and processed using the Fiji software package (Schindelin et al., 2012). Colors of images were adjusted (“false-colored”) to improve visibility, particularly for readers with red-green blindness. Axons were traced using the Simple Neurite Tracer plugin in Fiji (Schindelin et al., 2012), starting at the soma and using the smooth axonal morphology (in contrast to spiny dendrites) as a criterion. MSN density was analyzed in 40 μm sagittal sections containing Area X. For each bird, we analyzed two to four different sections of both hemispheres. Within those we counted the number of labeled neurons in at least eight stacks, each with the measures 100 × 100 × 8 μm and used the average of those to calculate density. We counted all nuclei (DAPI+) and all DARPP-32+ cells using the cell counter plugin in the Fiji software package (Schindelin et al., 2012).

### *In situ* hybridization

Hemispheres of birds used for *in situ* hybridization were separately frozen in Tissue-Tek O.T.C. Compound medium (Sakura) and stored at −80°C. Hemispheres were cut in 12 μm sagittal sections using a cryostat (Cryo-Star HM 560 Cryostat, MICROM). Sections were fixed with 4% PFA for 10 min and then acetylated with 0.25% acetic anhydride in triethanolamine for 10 min. Sections were rinsed in 2x in saline sodium citrate (SSC) buffer, dehydrated (75% EtOH, 95% EtOH, and 100% EtOH, each for 2 min) and air dried. Sections were prehybridized for 1 h at 60°C in a hybridization mix consisting of 50% deionized formamide, 5x SSC (pH 4.5), 2% blocking reagent (Roche, 11096176001) in 1x maleic acid buffer, 2% sodium dodecyl sulfate, yeast tRNA (Invitrogen, 0.25 mg/ml), and heparin (Polysciences, 0.1 mg/ml). Sections were hybridized overnight with 1% digoxigenin or fluorescin labeled RNA probe in hybridization mix at 60°C in a mineral oil bath. The next day, slides were rinsed twice with chloroform followed by 2x SSC and 1x SSC. A series of post-hybridization washes followed: 30 min in 1x SSC containing 50% formamide at hybridization temperature (60°C). Then, sections were washed once in 2x SSC and twice in 0.2x SSC 20 min each at hybridization temperature. After the post-hybridization washing steps, sections were washed twice in 1x MABT (pH 7.5), consisting of 100 mM maleic acid, 150 mM NaCl and 0.1% Tween-20. Afterwards, sections were incubated in 1x Roti-ImmunoBlock (Carl Roth) in 1x MABT for 30 min, then with either alkaline phosphatase (AP)-conjugated sheep anti-DIG antibody (Roche) or AP-conjugated sheep anti-fluorescein antibody (Roche), that were diluted 1:200 in 1x Roti-ImmunoBlock in 1x MABT. Slices were incubated overnight at 4°C in a humidity chamber. After antibody incubation, slides were washed with 1x MABT 4 times for 5 min and equilibrated in alkaline phosphatase buffer NTMT, consisting of 100 mM NaCl, 100 mM Tris hydrochloride pH 9.5, 50 mM MgCl_2_ and 0.1% Tween-20 for 10 min. AP-labeled probes were detected colorimetrically via the nitro blue tetrazolium/5-Bromo-4-chloro-3-indolyl phosphate substrate system (NBT/BCIP; Roche). NBT (final concentration: 337.5 μg/ml) and BCIP (final concentration: 175 μg/ml) were diluted in NTMT and slices were covered with this solution. Slices were incubated for 6–8 h, then fresh NBT/BCIP solution was added and sections were incubated overnight. The reaction was stopped by 10 min of incubation in a stop solution consisting of 10 mM Tris hydrochloride pH 8.0 and 1 mM EDTA. Afterwards, slides were washed three times with 1x PBS for 5 min. Sections were further used for immunohistochemical BrdU detection (see Immunohistochemistry) and examined with a Zeiss Axiovert 200 fluorescent microscope.

### Analysis and statistics

Data were analyzed with the data analysis software R (R Development Core Team, 2013) and GraphPad Prism version 5.00 (GraphPad Software, San Diego California USA). Data for EGR-1, DARPP-32 and DA receptor expression passed the D'Agostino's *K*^2^ test for normal distribution and were then evaluated with an analysis of variance (ANOVA) followed by a *post hoc* Tukey's Honestly Significant Difference test (HSD). To test the correlation between DARPP-32 density and age, we performed a linear regression analysis. Significance level was *p* < 0.05 for all tests.

## Results

### Newborn MSNs receive glutamatergic input and connect to pallidal output neurons

**Figure 2:**
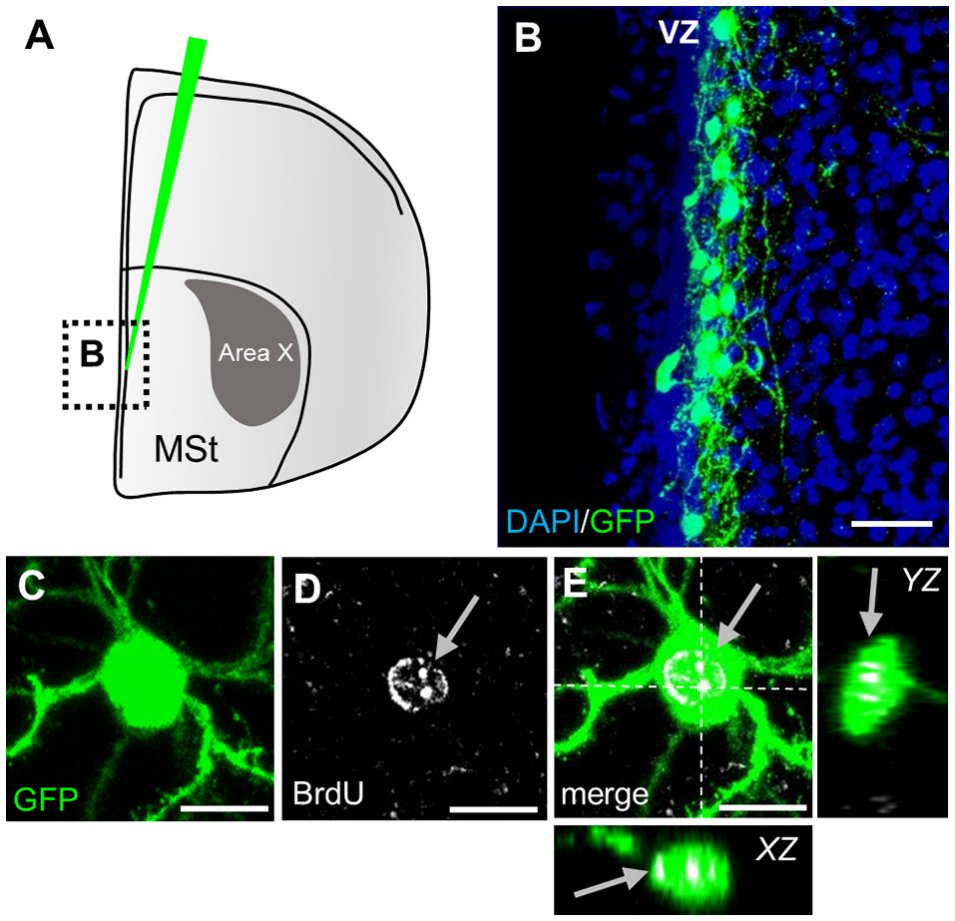
Labeling of striatal progenitors. **(A)** Lentiviral vector injections were surgically targeted at the wall of the lateral ventricle adjacent to the medial striatum (MSt). The area outlined is depicted in B. **(B)** Many cells in the ventricular zone (VZ) were infected, as shown by virally mediated GFP expression in a coronal section. The ventricle is on the left side of the image. **(C–E)** GFP+ neuron in the striatum recently divided and incorporated BrdU (arrows). Dashed lines **(E)** indicate the planes used to generate orthogonal views of the Z-stack (*YZ, XZ*). Scale bars: 25 μm **(B)**, 10 μm **(C–E)**.

To investigate whether and when newborn neurons in Area X are integrated into existing circuits, we used a lentivirally mediated approach to label progenitor cells in the striatal ventricular zone of adult male zebra finches (Figures 2A,B). By 31 days post injection (dpi), newly generated neurons in Area X exhibited the typical MSN morphology with relatively small nuclei (5–9 μm) and spiny dendrites. Co-labeling with BrdU confirmed that GFP+ cells in Area X recently divided and originated from the progenitor pool (Figures 2C–E).

**Figure 3:**
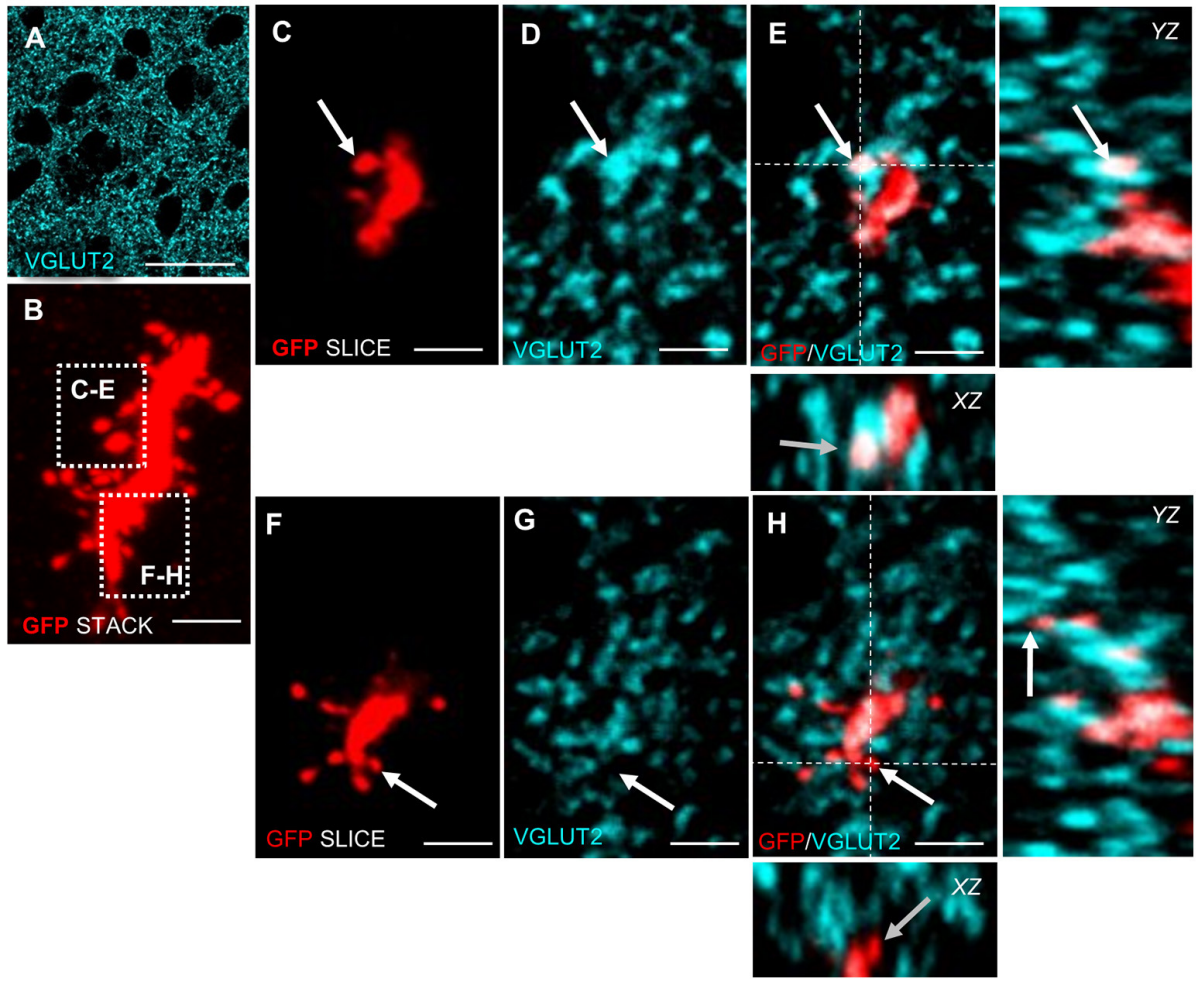
Adult generated MSNs in Area X receive glutamatergic input. **(A)** VGLUT2 is expressed in a punctate pattern in the neuropil, corresponding to presynaptic glutamatergic terminals in Area X. **(B)** High-resolution scan of an adult generated MSN dendrite (GFP+, red). The Z-scan was collapsed. The focus planes of spines in dashed boxes are shown in **C–H**. **(C–E)** Arrow points to a dendritic spine of an adult generated MSN that colocalized with VGLUT2. **(F–H)** Arrow points to a spine of new MSN that did not colocalize with VGLUT2. Dashed lines **(E,H)** indicate the planes used to generate orthogonal views of the Z-stack (*YZ, XZ*). Scale bars: 2.5 μm **(B–H)**, 25 μm **(A)**.

Newly generated granule neurons in the adult murine DG first receive long-range cortical inputs at 3 weeks of age, whereas granule cells in the olfactory bulb connect already at 2 weeks of age to presynaptic cortical neurons (Deshpande et al., 2013). We wanted to know if and when newborn MSNs in Area X receive glutamatergic inputs from afferent cortical song nuclei. Using VGLUT2 (vesicular glutamate transporter 2) (Figure 3A) as a marker we found glutamatergic synapses at spines of newly generated MSNs at 31 dpi (Figures 3B–E). These glutamatergic innervations are likely to originate from the pallial song nuclei HVC and LMAN (Figure 1). We also noticed spines without VGLUT2 immunoreactivity (Figures 3B,F–H).

**Figure 4:**
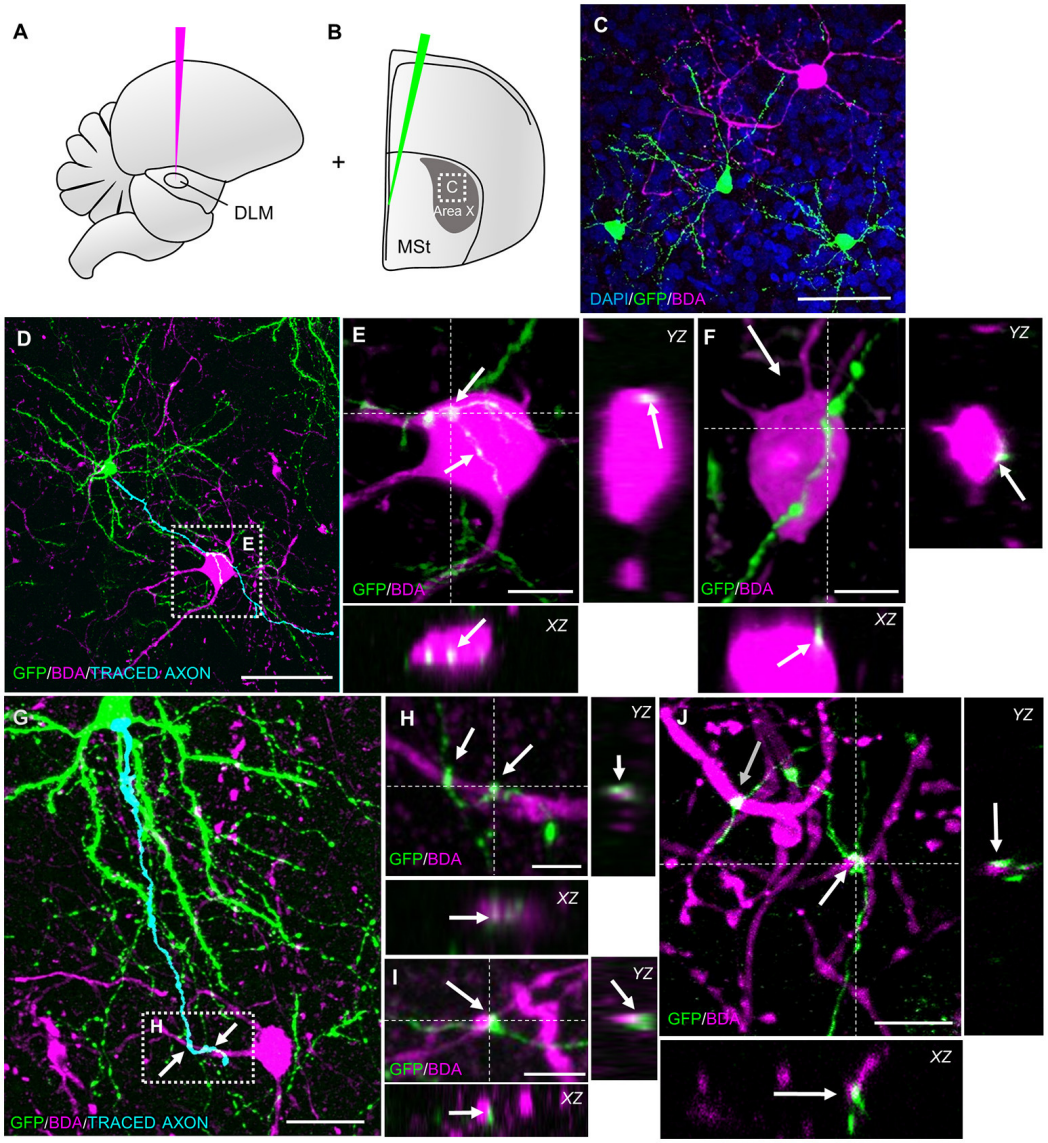
New MSNs have axosomatic and axodendritic contacts to pallidal-like projection neurons in Area X. **(A)** Pallidal-like projection neurons in Area X were labeled via retrograde tracing. BDA was injected into thalamic nucleus DLM, the target of pallidal-like projection neurons in Area X. **(B)** Additionally, progenitors were labeled in the VZ via lentivirally mediated GFP expression. The area outlined is depicted in **C**. **(C)** Newborn MSNs (GFP+) and pallidal-like neurons were both present in sections of Area X. **(D)** The axon of a newborn MSN passed the soma of a pallidal-like projection neuron (BDA). The pallidal-like neuron in the dashed box is magnified in **E**,**F**. **(E,F)** Axosomatic contacts (arrows) of new MSN on pallidal-like neuron somata. **(G)** The axon a newborn MSN wrapped around dendrites of a pallidal-like neuron. The area in the dashed box is magnified in **H**. **(H–J)** Axodendritic contacts (arrows) of newborn MSN onto pallidal-like neurons. Dashed lines **(E,F,H–J)** indicate the planes used to generate orthogonal views of the Z-stack (*YZ, XZ*). Scale bars: 5 μm **(H,I)**, 10 μm **(E,F,J)**, 25 μm **(G)**, 50 μm **(C,D)**.

After confirming glutamatergic input onto new MSNs, we tested if they contribute to signal transmission via pallidal-like output neurons. In the adult HVC, newborn projection neurons were found to be connected to their target nucleus at 3 weeks of age (Tokarev et al., 2016). We therefore predicted that newborn MSNs connected to their target cells in a similar way. Additional to GFP-labeling of progenitors in the VZ, we retrogradely labeled one class of pallidal-like neurons that project directly from Area X to the thalamic nucleus DLM (Figures 1, 4A,B; Goldberg et al., 2013). This neuron type is considered to be homologous to primate internal pallidal neurons (Goldberg and Fee, 2010). Retrogradely labeled neurons had big somata and smooth, aspiny dendrites; consistent with this cell type (Reiner et al., 2004; Figure 4C). We found connections from newborn MSNs to pallidal-like neurons at 31 dpi and 42 dpi. We observed connections between axons and axonal boutons of new MSNs and dendrites of pallidal-like neurons; in that case, axons often wrapped around pallidal-like neuronal dendrites (Figures 4G–J). Additionally, their axons were often found in close apposition to the somata of pallidal-like neurons (Figures 4D–F). We specifically searched for backfilled pallidal-like neurons with new MSNs (GFP+) nearby. At 31 dpi, we observed that in a fraction of 0.73 of pallidal-like neurons, new MSN axons contacted their dendrites. In a fraction of 0.27 of pallidal-like neurons, both their somata and dendrites received contacts by new MSNs axons (in total 22 pallidal-like neurons, 2 animals). At 42 dpi, we found that in a fraction of 0.69 of pallidal-like neurons, new MSN axons contacted their dendrites. In a fraction of 0.31 of pallidal-like neurons, both their somata and dendrites received contacts by new MSNs axons (in total 26 pallidal-like neurons, 2 animals).

### Newborn MSNs receive dopaminergic innervation

**Figure 5:**
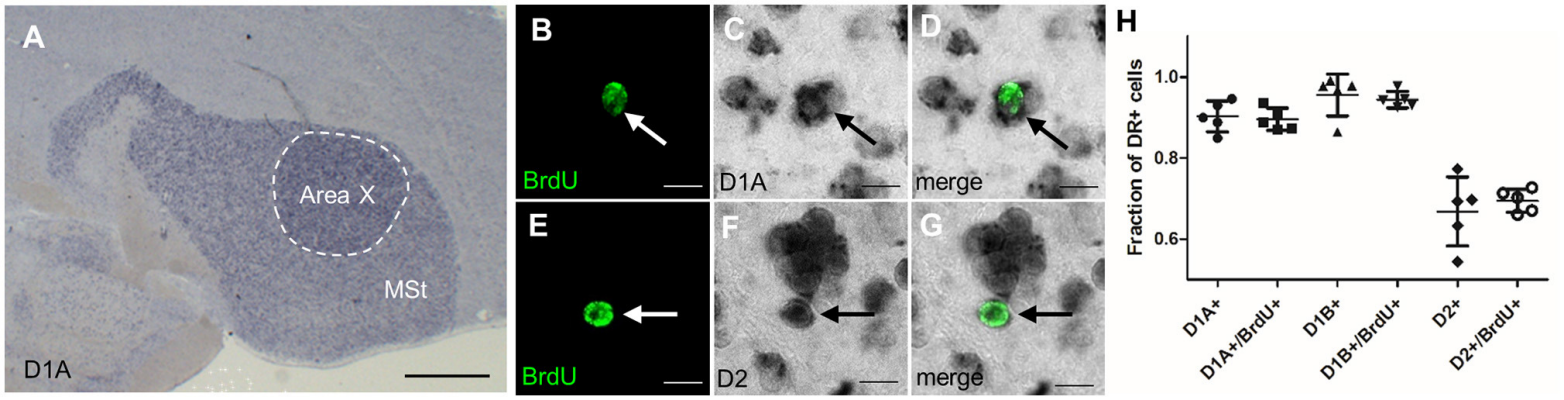
Newborn MSNs do not differ from mature MSNs in their DA receptor expression. **(A)** Dopamine receptors were highly expressed in the MSt and Area X shown here for D1A in a non-fluorescent *in situ* hybridization (blue precipitate). **(B–D)** New MSNs (BrdU+, arrow, fluorescent green label) expressed dopamine receptor D1A (dark precipitate). **(E–G)** New MSNs (BrdU+, arrow) expressed DA receptor D2. **(H)** There was no significant difference in the expression of dopamine receptor types between older neurons (BrdU-) and 42-day-old neurons (BrdU+). One data point represents one animal. Shown are mean and SD. Scale bars: 10 μm **(B–F)**, 500 μm **(A)**.

**Figure 6:**
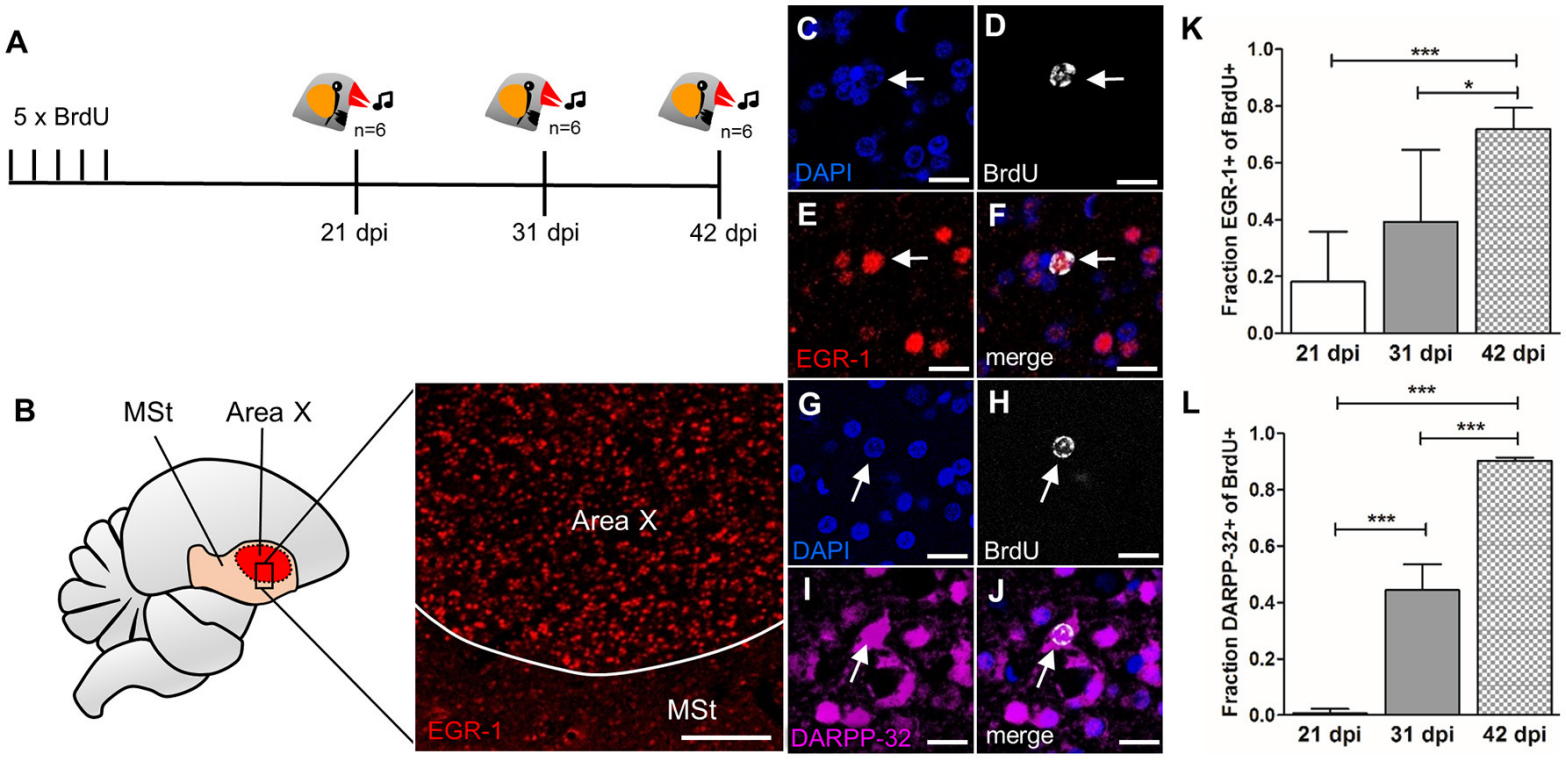
Newborn MSNs are activated during singing in an age-dependent manner. **(A)** Experimental design: Adult male zebra finches (*n* = 6 per age group) received BrdU injections and were sacrificed at 21, 31, or 42 dpi after singing. **(B)** Region specific EGR-1 expression in Area X but not in the surrounding striatum after singing. **(C–F)** Newborn neuron (31 dpi, arrow) expressed EGR-1 after singing. **(G–J)** Newborn neuron (31 dpi arrow) expressed DARPP-32. **(K)** The fraction of new cells (BrdU+) that were activated after singing (EGR-1+) increased significantly between 21 and 42 dpi and between 31 and 42 dpi (shown are mean and SD). **(L)** The fraction of new cells (BrdU+) that express DARPP-32 increased significantly from 21 to 42 dpi (shown are mean and SD). ^*^P ≤ 0.05; ^***^P ≤ 0.001. Scale bars: 10 μm **(C–J)**, 100 μm **(B)**.

Besides glutamatergic input from the song nuclei HVC and LMAN, MSNs in Area X also receive dopaminergic innervations from the ventral tegmental area (VTA) and the substantia nigra pars compacta (SNc), (Lewis et al., 1981; Bottjer, 1993; Gale et al., 2008). DA signaling via D1 receptors modulates social context dependent song variability; DA concentration in Area X is higher during female directed courtship song than when birds sing by themselves (Sasaki et al., 2006; Leblois et al., 2010). DA signaling can either be activating or inhibiting, depending on the receptor it is binding to Gerfen and Surmeier (2011). DA binding to D1-like receptors rises the resting potential and hence increases the chance of an action potential, whereas DA binding to D2-like receptors has the opposite effect. Neurons in the avian striatum express four types of dopamine receptors. Different from mice, up to 50% of MSNs in songbirds express both D1 and D2 receptor types (Kubikova et al., 2010). To test if newborn neurons differ from older, resident neurons in Area X in their expression of DA receptors, we combined *in situ* hybridization to detect DA receptor mRNA with BrdU labeling (Figures 5A–G). Because the majority of new neurons were mature at 42 days after BrdU labeling (Figure 6L), we decided to analyze DA receptor expression at that point. We found that a fraction of 0.89 ± 0.03 of new neurons expressed D1A, 0.94 ± 0.02 D1B, and 0.69 ± 0.03 D2 receptor mRNA (Figure 5H).

These results did not differ statistically from DA receptor mRNA expression values we found in non-BrdU labeled cells (0.9 ± 0.08 D1A, 0.95 ± 0.05 D1B, and 0.66 ± 0.08 D2). The averages of single-labeled D1A and D2 cells added up to more than 1, indicating that at least a fraction of 0.58 of BrdU+ cells co-expressed both receptor types. The averages of single-labeled D1B and D2 indicate that at least a fraction of 0.63 of BrdU+ cells co-expressed D1B and D2 receptors.

### Age dependent activation of newborn MSNs during singing behavior

Having confirmed that newborn MSNs receive both glutamatergic and dopaminergic input and are connected to output neurons, we tested if they participate in signal transduction during singing. We used the immediate early gene EGR-1 as an indicator for neuronal activity (Knapska and Kaczmarek, 2004) in Area X and quantified its expression after singing in new neurons at different survival times (Figure 6A). Undirected singing resulted in elevated EGR-1 expression in Area X (Figure 6B), as expected (Jarvis et al., 1998; Mello and Ribeiro, 1998).

The fraction of singing-activated, newborn neurons in Area X (BrdU+/EGR-1+, Figures 6C–F) cells increased from 0.18 ± 0.17 at 21 dpi to 0.72 ±0.07 at 42 dpi (*F* = 13.05, *p* = 0.00038, Figure 6K). There was no significant difference in activation of new neurons between 21 and 31 dpi (*F* = 13.05, *p* = 0.149), but between 31 and 42 dpi (*F* = 13.05, *p* = 0.019, Figure 6K). Additionally, we evaluated the maturation course and quantified the expression of the MSN marker DARPP-32 in newborn neurons (BrdU+/DARPP-32+, Figures 6G–J). DARPP-32 expression significantly increased from 0.0075 ± 0.015 at 21 dpi to 0.44 ± 0.09 at 31 dpi (*F* = 180.8, *p* = 6.3 × 10^−6^) to 0.9 ±0.01 at 42 dpi (*F* = 180.8, *p* = 8.3 × 10^−6^, Figure 6L).

### Age dependent MSN density in area X

When studying adult neurogenesis, it is always of concern whether newly-generated neurons are added continuously to an existing circuit or if they replace older neurons. Both strategies can occur in the same organism: newly generated granule cells in the mouse DG are added to the existing cell pool, whereas in the olfactory bulb new granule cells replace old neurons (Crespo et al., 1986; Imayoshi et al., 2008).

**Figure 7:**
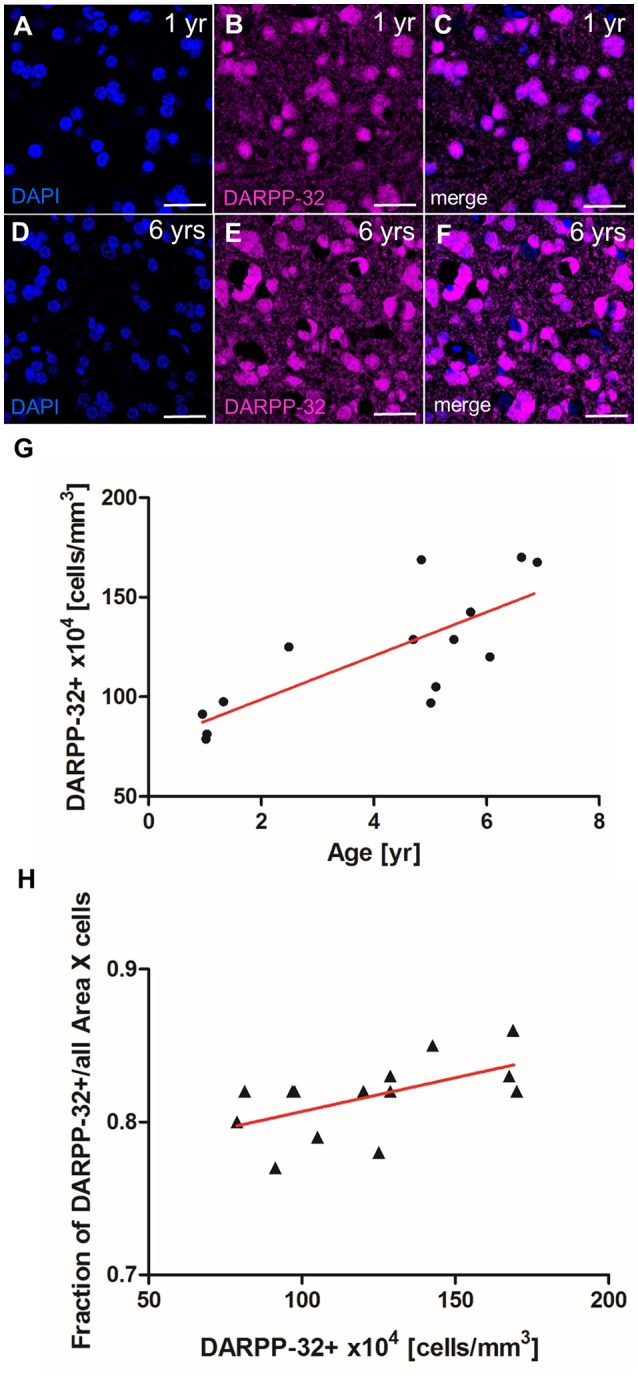
MSN density in Area X increases with age. (A–C) MSNs (DARPP-32+) in Area X of a 1-year-old zebra finch male. **(D–F)** MSN (DARPP-32+) in Area X of a 6-year-old zebra finch male. **(G)** The density of MSNs (Darpp-32+) increased significantly with age. **(H)** The fraction of MSNs (DARPP-32+) of all DAPI+ nuclei increased with MSN density. In **(G,H)** one data point represents one animal (mean of both hemispheres). Scale bars: 20 μm **(A–F)**.

In the canary song control nucleus HVC, newly generated projection neurons are replaced seasonally, while in the zebra finch HVC, new neurons are continuously added to the existing circuit (Walton et al., 2012). To investigate which strategy applies in Area X of zebra finches, we quantified the density of MSNs in zebra finches at different ages. MSN density in Area X increased significantly between 1 and 6 years of age (linear regression, *R*^2^ = 0.679 *p* = 0.0003, Figure 7G). MSN packing density increased from 78 × 10^4^ cells/mm^3^ in Area X of a 1-year-old zebra finch (Figures 7A–C) to 163 × 10^4^ cells/mm^3^ in as 6-year-old zebra finch (Figures 7D–F). Assuming an Area X size of 1.532 mm^3^ (Nixdorf-Bergweiler, 1996) the total number of MSNs in Area X more than doubled from 1.2 to 2.5 million within 5 years. The fraction of MSNs out of all DAPI+ cells also increased significantly with MSN density (linear regression, *R*^2^ = 0.34 *p* = 0.0286, Figure 7H).

## Discussion

In the present study, we investigated key features of adult-generated MSN that integrate into the avian striatal song nucleus Area X. Area X receives long-range cortical glutamatergic innervations from premotor nuclei HVC and LMAN (Bottjer and Johnson, 1997). We tested whether newborn MSNs in Area X receive this input by searching for glutamatergic presynaptic terminals on GFP-labeled newborn neurons after their migration from the ventricular zone. We found those contacts as early as 31 dpi. This time frame of being contacted by long-range excitatory input is similar to that reported for newborn hippocampal granule cells in mice (Deshpande et al., 2013), even though the migration distance of new MSNs from the VZ to Area X is considerably longer. This suggests that glutamatergic innervation of adult-born neurons is more a question of absolute age than a question of time of arrival at their final destination. We did not find presynaptic terminals on all dendritic spines, perhaps because those were in the process of being contacted or eliminated (Ramiro-Cortes et al., 2014).

Besides glutamate, dopaminergic innervation from VTA and SNc is the second main input to Area X (Lewis et al., 1981; Bottjer, 1993; Gale et al., 2008). By combining BrdU birth dating with *in situ* hybridization for DA receptors we established that 6-weeks old MSNs in Area X expressed mRNA for D1- and D2-type receptors in the same fractions as older, resident neurons. This suggests that newborn Area X neurons participate in dopaminergic signaling in the same way as older neurons do. It would be interesting to test if a time-dependent dopamine receptor expression in new neurons was crucial for specific stages of neurogenesis. For example, dopaminergic innervation via D3 receptors stimulates the very early process of progenitor proliferation in mammals and birds (Coronas et al., 2004; Lukacova et al., 2016) and in new murine granular cells, D1-type receptor expression is found earlier than D2-type receptor expression (Mu et al., 2011).

Having established the inputs onto new MSNs we were interested in their connection to pallidal-like projection neurons inside Area X. Direct pallidal-like neurons project to thalamic nucleus DLM and exhibit different firing patterns than indirect pallidal-like neurons (Goldberg and Fee, 2010; Woolley et al., 2014). We observed terminal boutons of newborn MSNs in close proximity to somata and dendrites of direct pallidal-like neurons. This suggests that newborn Area X neurons participate in signal transduction via the pallidal-like projection neurons. Future studies might address whether the innervation and connectivity to output neurons occurs even earlier than by 31 days after generation in the VZ, the time point we chose.

Given that newborn MSN have the morphological hallmarks to receive and transmit signals within Area X, we tested whether they are active during production of undirected song, which is known to induce EGR-1 protein expression (Jarvis et al., 1998; Mello and Ribeiro, 1998). We found that 20% of 21 day old MSN expressed EGR-1 after singing, but DARPP-32 was not detected in any MSN at that age. By 42 days of age, the majority of newborn MSNs expressed both proteins, raising the possibility that new MSNs may have to be physiologically active to trigger their further maturation. This is consistent with the fact that in mammals EGR-1 acts as a transcriptional activator of DARPP-32 (Keilani et al., 2012). One interpretation of our data is that singing-driven EGR-1 triggers maturation of newborn MSNs. This idea is supported indirectly; in mammals, the brain-derived neurotrophic factor (BDNF) enhances EGR-1 binding to the *Darpp-32* gene (Keilani et al., 2012). In canaries, BDNF levels are positively correlated with singing and enhance the survival of newly recruited HVC neurons (Rasika et al., 1999; Li et al., 2000). Similar mechanisms were shown in rodents; voluntary running exercise increases BDNF levels (Kobilo et al., 2011) and individual running activity positively correlates with rates of neurogenesis in the DG (Kodali et al., 2016). If overall individual singing activity influenced neuronal maturation via a BDNF/EGR-1/DARPP-32 pathway, it could explain the high variance in the fraction of activated new MSN during the early maturation phase (31 dpi) in contrast to the later maturation phase (42 dpi). New neurons that survived by then might have reached a stable state, whereas others that were not reliably EGR-1 activated by behavior were eliminated, similar to mechanisms found in the DG of mice (Veyrac et al., 2013).

Are new neurons in Area X added to existing circuits as a replacement of neurons that have died or are they added to the existing cell pool? In the songbird HVC both strategies exist: in canary HVC, seasonal fluctuations in projection neuron death and the recruitment of new neurons are correlated and the peaks of neural recruitment coincide with the incorporation of new song elements. Together these data are consistent with a replacement strategy (Kirn et al., 1994). In the zebra finch HVC, new projection neurons are added constantly to HVC, resulting in an increasing density within the nucleus (Walton et al., 2012). Correlative evidence suggests that the age-dependent decline of new neuron addition in HVC is associated with increasing song stereotypy (Pytte et al., 2007). Together, these data are best explained by an addition strategy. In the present dataset, we show that the density of DARPP-32 positive MSNs in Area X increased significantly with age, implying that new MSNs were constantly added to the circuit. This does not exclude the possibility that some new neurons replaced apoptotic cells. In fact, experimentally induced apoptosis correlates with replacement by new neurons in zebra finch HVC (Scharff et al., 2000). Further, we found that the fraction of cells that were DARPP-32+ relative to all Area X cells also increased with age. Since the DARPP-32 neurons constitute the majority of cells that undergo adult neurogenesis, this finding emphasizes that increased cell density in Area X is a consequence of continued recruitment of newly born MSN during the course of aging.

Our findings suggest that, once matured, newborn MSNs fulfill the same function as older, resident MSNs, at least concerning the features we analyzed. MSNs function via feed forward inhibition, e.g. sparsely spiking MSNs inhibit tonically active pallidal-like projection neurons. Their high frequency bursts can evoke spiking of DLM neurons via inhibitory rebound (Person and Perkel, 2005, 2007; Kojima and Doupe, 2009). This process in modulated by dopaminergic signals from VTA/SNc. Dopaminergic neurons in VTA/SNc encode performance errors in singing zebra finches (Gadagkar et al., 2016).

We end on some speculations how constant addition of new neurons might affect the AFP and in turn the motor pathway. Constant MSN addition in face of an unchanged number of pallidal-like neurons would be expected to cause stronger inhibitory MSN action on pallidal-like neurons. In turn, DLM would experience fewer inhibitory rebound spikes, causing lower activation of LMAN neurons. Ultimately this would result in reduced excitation of motor nucleus RA by the AFP. If this hypothesis holds true, signaling through the AFP would diminish, as birds get older. In adult birds, the AFP mediates differences in song variability (Hessler and Doupe, 1999; Woolley et al., 2014). Song variability, including deterioration, can be induced experimentally by distorting auditory feedback via deafening or tracheosyringeal nerve cut (Williams and McKibben, 1992; Hough and Volman, 2002; Nordeen and Nordeen, 2010). The AFP seems to mediate this degradation process, since lesions of the AFP output nucleus LMAN prevent song deterioration after auditory feedback distortion (Brainard and Doupe, 2000). Interestingly, song deterioration after deafening is less severe in old birds compared to young birds, and song becomes more stereotyped with age, consistent with our hypothesis (Lombardino and Nottebohm, 2000; Brainard and Doupe, 2001; Pytte et al., 2007, 2012). This scenario does not exclude the possibility that new MSNs initially might undergo a narrow plastic phase, during which they can be tuned and possibly counteract song drift. In summary, we demonstrate that within a month after their generation newly generated MSNs in Area X of adult zebra finches are connected to other song nuclei and participate in neuronal firing during song production. The net increase of Area X neurons with age might provide a mechanism to achieve the equilibrium between plasticity and stereotypy needed to sustain adult song behavior.

## Ethics statement

This study was carried out in accordance with the governmental law (TierSchG). The protocol was approved by the LAGeSo, Berlin.

## Author contributions

JK and CS planned experiments, JK and LS conducted experiments, JK and CS wrote the manuscript.

### Conflict of interest statement

The authors declare that the research was conducted in the absence of any commercial or financial relationships that could be construed as a potential conflict of interest.
